# Absence of Suction Feeding Ichthyosaurs and Its Implications for Triassic Mesopelagic Paleoecology

**DOI:** 10.1371/journal.pone.0066075

**Published:** 2013-12-11

**Authors:** Ryosuke Motani, Cheng Ji, Taketeru Tomita, Neil Kelley, Erin Maxwell, Da-yong Jiang, Paul Martin Sander

**Affiliations:** 1 Department of Earth and Planetary Sciences, University of California Davis, Davis, California, United States of America; 2 Department of Geology and Geological Museum, Peking University, Beijing, China; 3 Hokkaido University Museum, Hakodate, Japan; 4 Paläontologisches Institut und Museum, Universität Zürich, Zürich, Switzerland; 5 Steinmann Institute, Division of Palaeontology, University of Bonn, Bonn, Germany; University of Pennsylvania, United States of America

## Abstract

Mesozoic marine reptiles and modern marine mammals are often considered ecological analogs, but the extent of their similarity is largely unknown. Particularly important is the presence/absence of deep-diving suction feeders among Mesozoic marine reptiles because this would indicate the establishment of mesopelagic cephalopod and fish communities in the Mesozoic. A recent study suggested that diverse suction feeders, resembling the extant beaked whales, evolved among ichthyosaurs in the Triassic. However, this hypothesis has not been tested quantitatively. We examined four osteological features of jawed vertebrates that are closely linked to the mechanism of suction feeding, namely hyoid corpus ossification/calcification, hyobranchial apparatus robustness, mandibular bluntness, and mandibular pressure concentration index. Measurements were taken from 18 species of Triassic and Early Jurassic ichthyosaurs, including the presumed suction feeders. Statistical comparisons with extant sharks and marine mammals of known diets suggest that ichthyosaurian hyobranchial bones are significantly more slender than in suction-feeding sharks or cetaceans but similar to those of ram-feeding sharks. Most importantly, an ossified hyoid corpus to which hyoid retractor muscles attach is unknown in all but one ichthyosaur, whereas a strong integration of the ossified corpus and cornua of the hyobranchial apparatus has been identified in the literature as an important feature of suction feeders. Also, ichthyosaurian mandibles do not narrow rapidly to allow high suction pressure concentration within the oral cavity, unlike in beaked whales or sperm whales. In conclusion, it is most likely that Triassic and Early Jurassic ichthyosaurs were ‘ram-feeders’, without any beaked-whale-like suction feeder among them. When combined with the inferred inability for dim-light vision in relevant Triassic ichthyosaurs, the fossil record of ichthyosaurs does not suggest the establishment of modern-style mesopelagic animal communities in the Triassic. This new interpretation matches the fossil record of coleoids, which indicates the absence of soft-bodied deepwater species in the Triassic.

## Introduction

Many large predators in the modern marine ecosystem are mammals. A similar role was probably played by marine reptiles in the Mesozoic, until their last members became extinct 65.5 million years ago during the end-Cretaceous mass extinction [1], [2], leaving the niches open for marine mammals. However, it is not known how similar their feeding ecology was to that of modern marine mammals. Massare [3] was the first to identify feeding guilds among Mesozoic marine reptiles using tooth morphology and stomach contents. However, limited progress has been made in this field since her pioneering study, although some studies are being conducted [4], [5].

Ichthyopterygia must be considered when comparing modern marine mammals with Mesozoic marine reptiles because they were the Mesozoic analog of cetaceans. Being derived from four-legged reptiles, they evolved a fish-shaped body profile about 200 million years before cetaceans. Also, their fossil record is more complete than that of other marine reptile groups [6]. They were probably the most abundant marine reptiles in the Triassic and Jurassic seas, although they became extinct in the mid-Cretaceous [7]. They are the only group of Mesozoic marine reptiles for which deep diving has been proposed [8]. If an abrupt change in their feeding ecology is inferred from the fossil record, it probably reflects the changes in their prey community and its environment. Therefore, it is important to reconstruct the evolution of ichthyosaurian feeding ecology through geologic time.

Among the major questions in the evolution of feeding ecology in marine reptiles is the evolution of suction feeding. Suction feeding is the most common strategy for prey capture among extant marine vertebrates [9], yet its evolution among marine reptiles has attracted limited attention. Recently, it was suggested that Triassic shastasaurid ichthyosaurs contained three species of suction feeders based on their superficial resemblance to beaked whales (Ziphiidae) [10]. This expanded an earlier suggestion [11] that one of the three species, *Shonisaurus sikanniensis*, was a suction feeder analogous to beaked whales. If true, the feeding mode interpretation would have many ecological implications. Most importantly, it raises a question of whether a suitable environment for beaked-whale-type predators, including their prey community in the meso-/bathypelagic zones, was available in the Triassic. This question has remained unaddressed until now. Prior to these two studies, suggestions of suction feeding among Mesozoic marine reptiles were made for various stem sauropterygians including the placodont *Henodus* [12], and for the protorosaur *Dinocephalosaurus* [13]. *Henodus* is known only from the Upper Triassic of Germany in a lagoonal setting, whereas *Dinocephalosaurus* is a coastal species that is known only from the Middle Triassic of China. The other species are also coastal dwellers (*sensu* [14]) and have little relevance to mesopelagic ecology; therefore, they will not be mentioned hereafter.

The proposal of suction feeding in these shastasaurid ichthyosaurs was based on their resemblance to beaked whales, namely ‘short’ snout, tooth reduction, and ‘enlarged’ hyobranchial rod [10], [11]. These features were loosely defined and never tested quantitatively. However, a functional inference based on superficial resemblance, or “intuitive functional morphology” *sensu* [15], is often misleading. As with most functional questions, suction feeding in ichthyosaurs should be examined based on mechanical reasoning and quantitative tests. Re-evaluation of hyobranchial morphology is especially wanting because a recent study [16] showed that the hyobranchial size of *Guanlingsaurus* was likely overestimated by the previous study [10]. Also, new studies of hyobranchial structure in modern suction feeding vertebrates have been published since [10], e.g., [17], [18]. The hyobranchial apparatus is considered the most important hard-tissue correlate in the discussion of suction feeding in vertebrates [17]–[19] because it underlies the mechanism of subambient pressure generation for suction (see below).

The purpose of the present study is twofold: first to re-evaluate the hypothesis of suction feeding in ichthyosaurs through quantification of osteological features related to the mechanisms of suction; and second to discuss if a modern-style meso-/bathypelagic animal community existed in the Triassic and Early Jurassic, from the perspective of the large predators of the time. We will also review the feeding ecology of extant suction feeders among air-breathing marine vertebrates to make comparisons.

## Materials and Methods

### Data Collection

**Figure 1:**
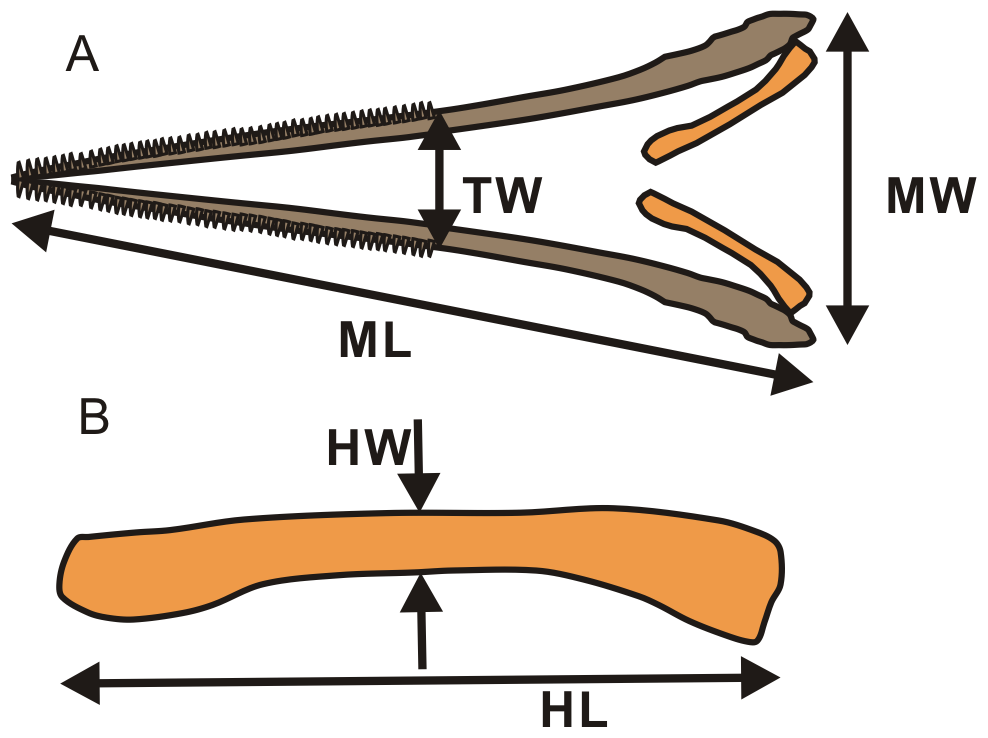
Measurements taken from ichthyopterygian specimens. a, schematic drawing of mandible with a pair of ceratobranchial I. b, magnified view of a single ceratobranchial I. HL: hyobranchial rod length; HW, hyobranchial rod median width; ML, mandibular ramus length; MW, mandibular width; TW, mandibular width at the end of tooth row. Brown, mandible; orange, hyobranchial rod.

We measured 18 ichthyosaurian specimens in which at least one hyobranchial rod is preserved. The small sample size is a consequence of the scarcity of such specimens. The samples span 15 genera and 18 species. They are *Chaohusaurus geishanensis* (AGM, Anhui Geological Museum, Hefei, China CH-628-22), *Eurhinosaurus longirostris* (SMNS, Staatliches Museum für Naturkunde, Stuttgart, Germany 57922), *Guanlingsaurus liangae* (Guanling National Geopark, Guanling, China dq-50), *Guizhouichthyosaurus tangae* (Wuhan Institute of Geology and Mineral Resources of China, Wuhan, China TR00001), *Hauffiopteryx typicus* (SMNS 81962), *Ichthyosaurus communis* (NHMUK, Natural History Museum, London, UK 36256), *Leptonectes moorei* (NHMUK R14370), *L. tenuirostris* (NHMUK 24300), *Mixosaurus cornalianus* (Paläontologisches Institut und Museum der Universität, Zurich T2414), *Qianichthyosaurus zhoui* (Institute of Vertebrate Paleontology and Paleoanthropology, Beijing, China 11838), *Shastasaurus alexandrae* (UCMP, University of California Museum of Paleontology, Berkeley, US 9017), *Shonisaurus sikanniensis* (Royal Tyrrell Museum of Paleontology, Drumheller, Canada 94.378.2), *Stenopterygius quadriscussus* (SMNS 50165), *St. triscissus* (SMNS 55074), *Suevoleviathan integer* (SMNS 4652), *Temnodontosaurus platyodon* (Oxford University Museum, Oxford, UK J29170), *Te. trigonodon* (SMNS 50000), and *Toretocnemus zitteli* (UCMP 8099). All but one specimen were in the existing collection of each institution and studied on-site in respective collections with permission. AGM CH-628-22 was excavated by a joint team of AGM, Peking University, University of California, Davis, and Università degli studi di Milano, with permits from the Ministry of Land and Resources of the People's Republic of China. The current study did not involve any purchase, loan, or donation of a specimen. Of the 18 specimens, mandibular width could reasonably be estimated in 15, and mandibular length in 12 specimens (Table 1)–mandibular rami were sometimes splayed beyond their natural angle and it was necessary to estimate the original width using the skull width when possible. Measurements taken are: hyobranchial rod length (HL), hyobranchial rod width at mid length (HW), mandibular ramus length (ML), mandibular width (MW), and mandibular width at the end of tooth row (TW). Fig. 1 summarizes the measurements taken, and Table 1 their values. TW for edentulous taxa was estimated by the mandibular width at the anterior margin of the orbit. This scheme may lead to a slight underestimation of the true values if applied to tooth-bearing taxa, and slight overestimation of suction feeding ability. However, such a bias does not affect the outcome of the study. Digital calipers were used for most measurements, which were recorded to the nearest 0.1 mm. Larger measurements were made by large calipers or tape measures and recorded to the nearest 1 mm. The mechanical importance of these measurements will be discussed later.

**Table 1:** Measurements (in mm) of ichthyopterygian mandible and hyobranchial rod.

	ML	MW	HL	HW	TW
*Chaohusaurus geishanensis*	103.4	—	11.7	1.3	—
*Eurhinosaurus longirostris*	650	200	143.0	13.1	45.0
*Guanlingsaurus liangae*	—	230	108.9	7.1	115.5
*Guizhouichthyosaurus tangae*	877	—	130.9	10.5	—
*Hauffiopteryx typicus*	—	134.0	98.0	9.0	—
*Ichthyosaurus communis*	376	83.1	88.1	6.0	44.2
*Leptonectes tenuirostris*	549	164.8	77.4	6.8	86.3
*Leptonectes moorei*	335	132.3	87.9	9.0	49.8
*Mixosaurus cornalianus*	184	—	30.0	2.5	—
*Qianichthyosaurus zhoui*	223	100.6	61.0	5.2	41.9
*Shastasaurus alexandrae*	—	205	227	16.7	—
*Shonisaurus sikanniensis*	—	1451	1220	78.8	1365
*Stenopterygius quadriscissus*	547	124.6	81.0	9.9	17.3
*Stenopterygius triscissus*	426	130.0	63.6	8.4	17.2
*Suevoleviathan integer*	537	192.9	103.2	9.9	104.1
*Temnodontosaurus platyodon*	1333	577	265	23.6	401
*Temnodontosaurus trigonodon*	—	420	365	21.9	190
*Toretocnemus zitteli*	—	82.3	57.4	5.2	35.5

See Fig. 1 for abbreviations and text for specimen number.

Data for sharks were taken from [18], which used CT scans of sharks for measurements. Cetacean hyobranchial metrics were derived from [17], whose measurements were taken from dry specimens. Mandibular measurements of extant marine tetrapods were taken from [4].

### Identity of Ossifications in Ichthyosaurian Hyobranchial Apparatus

The hyobranchial apparatus is usually a composite of hyoid and branchial elements, some of which are ossified/calcified while the rest are not. In mammals and sharks, the hyoid part is more dominant than the branchial part whereas in reptiles, the branchial part tends to be more dominant [18], [21], [30]. The apparatus comprises a median body (hyoid corpus) and at least one pair of horns (hyobranchial cornua). Each horn often has multiple segments, some of which may be ossified/calcified. There is usually a pair of ossified/calcified horn segments that is dominant within a given hyobranchial apparatus, and it tends to play a major role in the suspension of the entire apparatus. We will refer to these dominant horn segments as hyobranchial rods in this contribution.

The hyobranchial apparatus of ichthyosaurs is usually preserved as a pair of curved hyobranchial rods. These rods have variously been referred to as ceratohyals or hyoid rods [20], hypohyals [11] or simply hyoids, e.g., [6]. In the present contribution, the ossified rod is identified as the first ceratobranchial (CB1), given that when there is only one pair of rods ossified in the hyobranchial apparatus of extant reptiles, it is usually considered CB1 [21] in squamates [22], [23], crocodylians [24], and turtles [25]; the homology of the elements with those of fish, however, has been questioned [26].

Functionally, ichthyosaurian CB1 probably played two important roles. Given its position, it is likely that CB1 was involved in the suspension of hyobranchial apparatus, with its postero-lateral margin fixed, maybe via soft tissue, to the otic region of the cranium. The identity of the hyobranchial rod differs from clade to clade–it is the stylohyal in cetaceans, CB1 in reptiles, and the ceratohyal in sharks. We therefore compare the morphology of these hyobranchial rods across taxa because of functional similarity in suspension although they are not developmentally homologous with each other. CB1 of ichthyosaurs likely had other functions in addition to suspension: it most likely provided attachment surfaces for muscles that linked between the tongue anteriorly (M. hyoglossus, M. geniohyoideus, and M. mandibulohyoideus) and shoulder girdle posteriorly (M. sternohyoideus and M. omohyoideus), as in many extant reptiles [19], [27]–[29]. A similar role is played by thyrohyals in odontocete cetaceans [30], [31], although the stylohyal also provides attachment for an extrinsic tongue muscle (M. styloglossus). In sharks, it is the basihyal that provides attachment for the muscle to the shoulder (M. coracohyoideus) [32], whereas sharks generally do not have a muscular tongue [33].

### Definition of Suction Feeding and ‘Ram Feeding’

Despite the prevalence of suction feeding among aquatic vertebrates [9], the definition of the term “suction feeding” has been taxon-dependent and variable (Table 2), preventing broad comparisons across major vertebrate groups, such as sharks and cetaceans. When a jawed vertebrate (gnathostome) opens its mouth in water, a pressure gradient is necessarily produced, usually leading to subambient pressure in the mouth [34], [35]. Therefore, some level of ‘suction’ pressure is incurred during feeding in virtually all aquatic gnathostomes. However, the term suction feeder *sensu stricto* is applied only to those aquatic gnathostomes that draw prey closer by suction prior to capture. In contrast, those predators that capture their prey mainly by moving their body toward prey are called ‘ram feeders’ [36], even when the pressure gradient around the mouth helps the prey from being pushed away [34], [37].

**Table 2:** Taxon dependence of definition of suction and ‘ram’ feeding.

Taxon	Ref.	Suction moves prey toward predator for capture	‘Suction’ re-orients and transports prey in predator's mouth	Predator moves toward prey for capture
Teleost Fish	37	suction feeding	na	‘ram feeding’
Shark	53	suction capture	suction transport	ram feeding
Turtle	34	suction feeding	na	‘ram feeding’
	45	inertial suction	na	compensatory suction
Whale	43	suction feeding		raptorial feeding
	17	capture suction feeding	combination feeding	raptorial feeding
Comparative	This study	suction feeding	na	‘ram feeding’

Suction is usually used in combination with various degrees of ram feeding [38]–[40]. The relative contribution of suction versus ram during feeding is often quantified by the Ram Suction Index (RSI), which compares the relative contribution of prey versus predator movements in regard to the total distance of movements by the two [41]. Despite its weaknesses, e.g., [39], [42], use of the RSI is common in the study of feeding kinematics in chondrichthyans, osteichthyans, and chelonians.

Suction feeding in the literature on whales has been defined less strictly: it variously contains suction feeding *sensu stricto* as defined above, as well as feeding where prey is captured by ram, followed by intraoral transpiration of prey for swallowing with help from subambient pressure induced by tongue movements [43]. This broader definition, however, obscures the status of suction feeding in cetaceans because it contains two mechanisms of prey capture. It also prevents comparisons with suction feeders among other major vertebrate groups.

Recent kinematic studies are shedding light on the status of cetacean suction feeding in comparison to that in other major vertebrate groups. A study of the feeding kinematics of the pygmy and dwarf sperm whales (*Kogia sima* and *K. breviceps*) and bottlenose dolphin (*Tursiops truncatus*) found that *Kogia* was a suction feeder and *Tursiops* a ‘ram feeder’ based on RSI [44]. The average suction distance was negative for *Tursiops* (i.e., the prey was pushed away, indicating that it barely used suction for prey capture). Also, an analysis of the feeding kinematics of three species of cetaceans, namely the beluga whale (*Delphinapterus leucas*), Pacific white-sided dolphin (*Lagenorhynchus obliquidens*), and long-finned pilot whale (*Globicephala melas*) found that all were capable of drawing prey toward them through suction by about 2.8 to 4.1 cm, whereas these cetaceans also used various degrees of ‘ram feeding’ in combination with suction [35]. In terms of RSI values, only *Delphinapterus* had feeding sequences that qualified as suction feeding, although many of the feeding sequences of the same species represented ‘ram feeding’.

The use of the word ‘ramming’ for the movement of the predator toward its prey during capture is not universal across taxa (Table 2). Despite its use in a part of the cetacean literature [35], [44], it has been suggested that it is inappropriate to use this term for vertebrates without gills given its derivation [45], [46]–the term ‘ram feeding’ was first used in reference to ram ventilation, where water runs through the gills of certain fishes through swimming, rather than pumping. In this paper, we use the term ‘ram feeding’ to describe any feeding mode where movement of the body or jaws is mainly used to overtake prey, in order to permit a simple comparison across major vertebrate groups.

### Statistical Analyses

We used R 2.15.2 for all calculations. Bivariate regressions are based on Model II regression [47], which is also called Standardized Major Axis (SMA) regression. The package smatr [48] of R was used for this purpose. All variables were log-transformed before statistical analyses to account for scaling effects. All ratios were log-transformed because ratios between two normally distributed variables with different means are highly skewed. Analysis of covariance (ANCOVA) is based on the aov function of R.

## Mechanically Important Hard-Tissue Features

### Hyoid Corpus Ossification/Calcification

**Figure 2:**
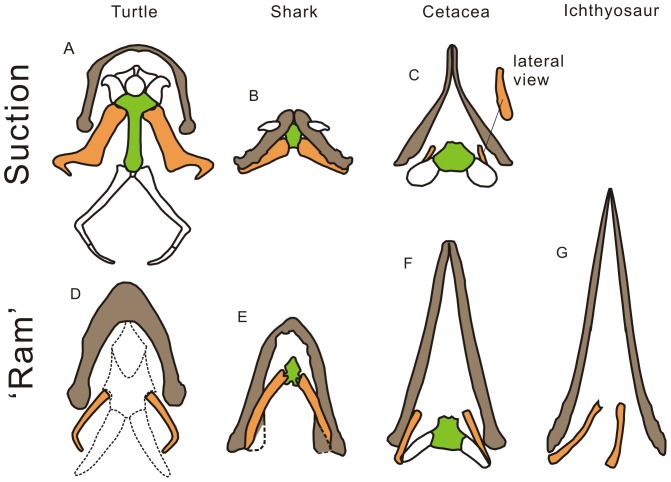
Mandible and hyobranchial apparatus of selected vertebrate groups. Suction feeders are in the top row, and ‘ram’ feeders bottom row. Brown fill, mandible; green fill, hyoid corpus; orange fill , ossified/calcified hyobranchial rods that are discussed; white fill with real outline, other ossified hyobranchial elements; white fill with dotted outline: cartilaginous hyobranchial element. Taxa: a, Mata Mata Turtle *Chelus fimbriatus*; b, Japanese Angel Shark *Squatina japonica*; c, Pygmy Sperm Whale *Kogia breviceps*; d, Common Musk Turtle *Sternotherus odoratus*; e, Sharpnose Sevengill Shark *Heptranchias perlo*; f, Bottlenose Dolphin Tursiops truncatus; and g, Triassic Ichthyosaur *Qianichthyosaurus zhoui*. Derivations: a based [19]; b and e from CT data; c and f based on [52]; d based on [24]; and g based on IVPP 11838. Not to scale.

The mechanism to produce subambient pressure in the oral cavity for suction feeding is almost uniform among jawed vertebrates, that is, posterior retraction and depression of the hyobranchial apparatus allowing expansion of the volume of the pharyngeal region, e.g., [18], [19], [49]. The muscles that connect the apparatus to the shoulder girdle, such as M. coracohyoideus (via M. coracoarcualis) or M. sternohyoideus, are recruited during the retraction, and the stress is applied to components of the apparatus, especially the central element (hyoid corpus) and the cornua that suspend the apparatus from the cranium (CB1 in reptiles, ceratohyal in sharks, and stylohyal in mammals). This common mechanism poses similar mechanical constraints on the morphology of the hyobranchial apparatus across taxa, allowing inference of suction capability based on hyobranchial morphology. One such mechanism-related feature is found in the hyoid corpus, or the central element of the hyobranchial apparatus. Suction-feeding jawed vertebrates have an ossified hyoid corpus that rigidly integrates with the right and left hyoid horns, in whales [50], turtles [19], [24], [42], [51], and sharks (TT pers. obs.; see also Fig. 2). It was specifically stated [24] that the absence of an ossified hyoid corpus in *Sternotherus* impaired the suction ability of this turtle. This is mechanically expected because the hyoid corpus is the central element of the hyobranchial apparatus that maintains its integrity and rigidity.

### Hyobranchial Rod Robustness

The second feature that is rooted in the same mechanical constraints concerns the hyobranchial cornua, especially the suspensory element. The suspensory ‘rods’ of suction-feeding jawed vertebrates are known to be more robust compared to those of closely-related ‘ram feeders’ [17]–[19], [42], [52]. This observation is deeply rooted in suction mechanics because, as pointed out earlier, suspensory hyobranchial elements of suction-feeding jawed vertebrates need to withstand the stress incurred by abrupt rotation that ultimately results from posterior pulling by M. coracohyoideus or M. sternohyoideus muscles during suction pressure generation [18], [19]. It should be noted that this value is biased by change in the degree of ossification/calcification through ontogeny. Juvenile CB1 of ichthyosaurs, for example, is expected to have had cartilaginous ends that are not preserved in fossils in addition to the ossified center part that is preserved. As a result, the CB1 of juveniles may appear more robust in fossils than it was in life. We tried to avoid this bias by measuring mature specimens; however, it was not possible for taxa that are known only from juvenile specimens. However, the effect of this bias is probably small, judging from the uniformity of CB1 robustness across ichthyosaurs that is reported later in this paper.

### Mandibular Pressure Concentration Index

**Figure 3:**
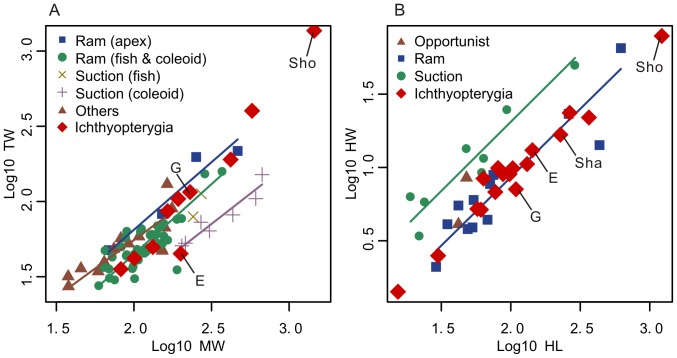
Bivariate SMA regression of features related to Mandibular Pressure Concentration Index and Hyobranchial Robustness. a. Mandibular Pressure Concentration Index; b. Mandibular Robustness. Lower intercept values in a and b indicate: a, higher pressure concentration within the oral cavity; and b, less robust suspensory element of hyobranchial apparatus. Ichthyopterygians has limited pressure concentration (high intercept in a) and slender hyobranchial rod (low intercept in b). E, G, Sha, and Sho denote *Eurhinosaurus*, *Guanlingsaurus*, *Shastasaurus*, and *Shonisaurus*, respectively. Note that the latter three were considered suction feeders by [10].

Subambient pressure produced in the oral cavity is projected beyond the gape plane to suck prey toward the predator. The propagation of pressure beyond the gape needs to be controlled so that it is concentrated and directed toward the prey [37], [49]. It has been pointed out that shorter and smaller gapes perform better in achieving this goal. Many teleost fish, for example, have mouths that can be protruded to form a small semi-circular margin with minimal gape incision that enables concentrated projection of pressure [37]. The suction system may be compared to a syringe, with the mouth opening corresponding to its aperture and the hyobranchial apparatus to the cylinder. Given that the square of the ratio between aperture and cylinder diameters of a syringe describes the pressure concentration that occurs between the pressure generator (cylinder) and propagator (aperture), a similar ratio for the predator's feeding system may be used to approximately describe the pressure concentration that occurs during suction. One way to calculate such a ratio is to divide the mandibular width at the jaw articulation by that at the end of the toothrow, the former approximating the width of hyobranchial apparatus, and the latter the mouth width at the gape angle. This ratio is here called the Mandibular Pressure Concentration Index (MPCI). MPCI is expected to be higher in suction feeders than in ‘ram feeders’. Measurements from extant marine mammals confirm this expectation (Fig. 3A).

### Mandibular Bluntness

In odontocete cetaceans, it was found that shorter snouts were better suited for concentrating pressure projection based on measurements of suction pressure from the heads of three odontocete species with varying snout lengths [49]. For this mechanical reason, it is expected that mandibular bluntness [17], [49] is a feature of suction-feeding jawed vertebrates. It should be noted that mandibular bluntness, as measured by mandibular width/mandibular ramus length, has its limitation for being a simple ratio. Mandibular width exhibits a negative interspecific allometry against mandibular ramus length; therefore the larger the animal, the less blunt the mandible. However, given that the cetaceans and ichthyosaurs in question span similar size ranges, the range of mandibular bluntness in each group is still a useful indicator of average suction ability.

### Features Not Considered

**Figure 4:**
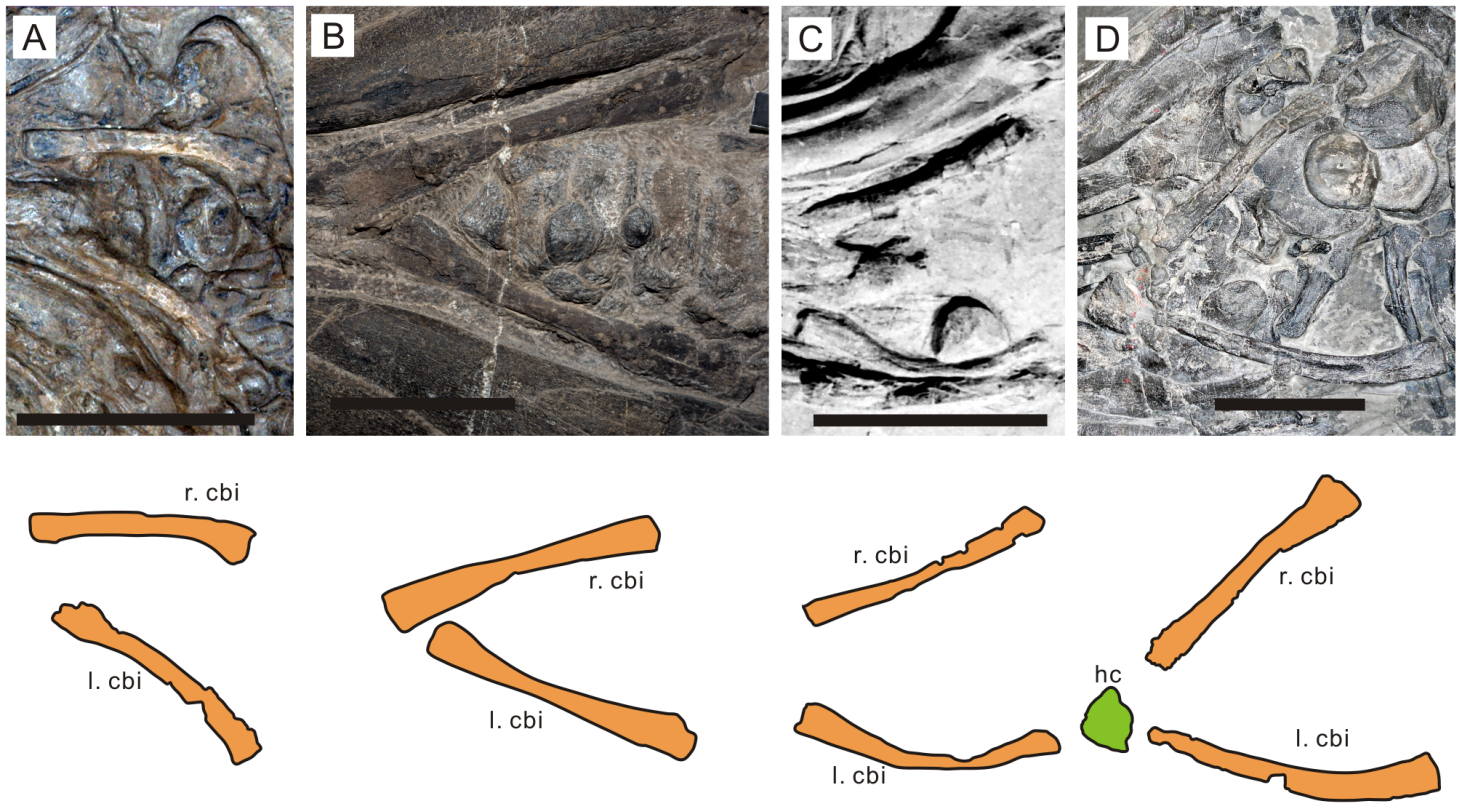
Hyobranchial apparatus of selected ichthyopterygians. a., *Qianichthyosaurus zhoui* (IVPP 11838); *Guanlingsaurus liangae* (GNG dq-50);c, *Ichthyosaurus communis* (OUM J10313); and d, *Hauffiopteryx typicus* (SMNS 81962). cbi, ceratobranchial I; hc, hyoid corpus; l, left; r, right. Scale bars are five centimeters.

Tooth reduction is another feature that has been discussed in the literature on whales [17], [43] and sharks [53]. The word ‘reduction’, however, is used with different connotations in the two clades. In sharks, it is the reduction of size, as seen in the bottom-feeding nurse shark (*Ginglymostoma cirratum*), whitespotted bamboo shark (*Chiloscyllium plagiosum*), and the planktivorous whale shark (*Rhincodon typus*) [53], [54]. In whales, reduction usually refers to the reduction of tooth count [43], which may or may not be associated with decrease in tooth size. Therefore, it is difficult to define the term uniformly across taxa. To complicate the problem further, Werth [43](fig. 4) showed that tooth count reduction was clearly correlated with shortening of the mandible; this is expected because shorter mandibles have less space for teeth. Given that mandible bluntness has a hydromechanical reason to affect suction ability, this correlation has to be removed before the contribution of tooth reduction to suction ability can be tested. Also, extreme reduction in tooth count is only known among beaked whales (apart from *Tasmacetus*), mysticetes, and in *Monodon*, whose suction feeding has not been directly observed (i.e., a large contribution of ‘ramming’ may be possible, as in *Globicephala* and most feeding sequences of *Delphinapterus*). The two cetacean genera for which strict suction feeding has directly been established through kinematic studies (*Kogia* and some feeding sequences of *Delphinapterus*) have multiple pairs of teeth at least in the lower jaws. Furthermore, filter feeding mysticetes also exhibit completely reduced dentition; however only one mysticete, the gray whale *Eschrichtius robustus*, is a reported suction feeder [55], [56]. Thus tooth reduction alone is not an unequivocal indicator of suction feeding.

## Results

### Hyoid Corpus Ossification/Calcification

The fossilized hyobranchial apparatus of ichthyosaurs comprises a pair of CB1, whose posterior ends are located near that of the mandible in virtually all specimens examined, although the bones were preserved more anteriorly or posteriorly in some specimens than in others (Fig. 4). The only exception is *Shonisaurus sikanniensis*, which seems to have a small second pair of bones [11]. There was no evidence for ossified hyoid corpus in all but one specimen examined. The exception is *Hauffiopteryx* from the Toarcian (Lower Jurassic), in which a bone exists in front of the pair of CB1 (Fig. 4D). Given its symmetrical shape and position, it is tentatively identified as the hyoid corpus. As preserved, there is no evidence to suggest that the hyoid corpus and CB1 were strongly integrated in this taxon. The preservation suggests that a large part of the hyobranchial apparatus remained cartilaginous in ichthyosaurs, as in many reptiles [20].

### Hyobranchial Rod Robustness

The shape of CB1 varies from strongly curved to almost straight, probably reflecting different degrees of compression during preservation rather than taxonomic differences. The effect of compaction is especially evident in one specimen of *Ichthyosaurus* (Fig. 4C), where the right CB1 is straight while the left one is curved. Virtually all CB1 were expanded toward both ends, with a slight constriction near the mid-shaft region. The anterior expansion is usually larger than the posterior one when the bone is well preserved, but there were some exceptions depending on the angle of fossil exposure. Many of them were somewhat sigmoidal in shape.

**Figure 5:**
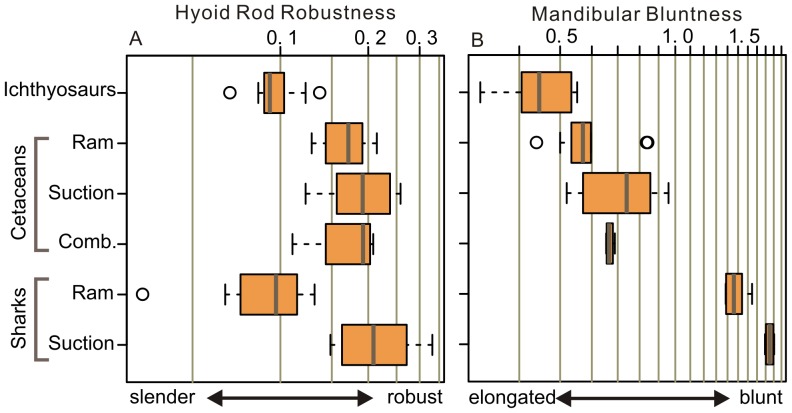
Boxplots of two ratios across taxa and feeding types. a, hyobranchial robustness; b, mandibular bluntness. The thick line in the center denotes the median, the box surrounding it contains the middle 50% of the data points, and the whiskers extend to the most extreme data point which is no more than 1.5 times the interquartile range from the box. The data outside the whisker are considered outliers, plotted as small circles.

The CB1 of ichthyosaurs are more slender compared to suspensory hyobranchial elements of suction-feeding turtles (CB1), sharks (ceratohyal), and cetaceans (stylohyal)(Fig. 2; Fig. 5A). This can be quantified by comparing the mid-shaft diameter and length (Fig. 3B), although this ratio scales with size to some extent, revealing weakly negative interspecific allometry with a slope of 0.93 (Fig. 3B). It was also observed that the slenderness of the ichthyosaurian CB1 is similar to that of ceratohyals in ‘ram-feeding’ sharks (Fig. 3B). Notably, ichthyosaurian CB1 slenderness does not vary as widely as in sharks (Fig. 3B), suggesting that the mechanical function of the hyobranchial apparatus was largely uniform among ichthyosaurs. ANCOVA revealed a significant difference between regressions for suction-feeding sharks and ichthyosaurs (p = 9.60×10^−8^, df = 1, F = 62.78) but not between those of ‘ram-feeding’ sharks and ichthyosaurs (p = 0.777, df = 1, F = 0.082). ‘Ram-feeding’ cetaceans have more robust suspensory hyobranchial elements than do ‘ram-feeding’ sharks or ichthyosaurs (Fig. 5A). This anomaly, however, can be explained by suction feeding being plesiomorphic for cetaceans [17]. The close similarity in robustness of hyobranchial rods across clades is surprising given the expected difference in material properties of calcified cartilage of sharks and bones of tetrapods. Note, however, that the hyobranchial rod is slightly more slender in ichthyosaurs than in ‘ram-feeding’ sharks based on the median value, possibly reflecting the difference in material property. A careful literature search for the material property of calcified shark cartilage proved unsuccessful, and it is currently difficult to investigate this aspect.

### Mandibular Pressure Concentration Index

The MPCI of ichthyosaurs has a small variation, but it fits in the range of ‘ram-feeding’ marine mammals, with one exception of *Eurhinosaurus* (Fig. 3A). This observation is supported by ANCOVA: when plotting the mandibular width at the end of the tooth row against the maximum mandibular width as in Fig. 3A, there was a significant difference between regressions of suction-feeding marine mammals and ichthyosaurs excluding *Eurhinosaurus* (p = 4.57×10^−3^, df = 1, F = 10.9), but not between those of ‘ram-feeding’ marine mammals and ichthyosaurs (p = 0.122, df = 1, F = 2. 46).

### Mandibular Bluntness

Ichthyosaurs are generally longer-snouted and therefore have low mandibular bluntness compared to odontocete cetaceans or sharks (Fig. 5B). Even the shortest-snouted ichthyosaurs in our data set, such as *Qianichthyosaurus* and *Guanlingsaurus*, have mandibular bluntness of about 0.42 or lower. These low bluntness values are found only in some beaked whales among suction-feeding odontocetes, whereas all others have much higher bluntness (Fig. 5B). Note that relatively low mandibular bluntness of beaked whales is compensated for by the precoronoid crest of the mandible that shortens the effective gape size.

## Discussion

### Possibility of Suction Feeding

The hyobranchial apparatus of Triassic and Early Jurassic ichthyosaurs strongly suggests that these marine reptiles were incapable of suction feeding. All but one species (*Hauffiopteryx typicus*) lacked an ossified hyoid corpus, whereas robust integration between ossified hyoid corpus and cornua has been identified as essential for amniote suction feeders as pointed out earlier. Even in *H. typicus*, such a robust integration is questionable (Fig. 4D). Also, the CB1 of ichthyosaurs, including *H. typicus*, is not very robust compared to suspensory hyobranchial elements of suction-feeding turtles (CB1), whales (stylohyals), and sharks (ceratohyals) (Figs. 2, 3, and 5). Therefore, it seems reasonable to conclude that all ichthyosaurs examined were ‘ram feeders’ because they lacked hyobranchial adaptation toward suction feeding. Notably, two of the presumed suction feeders [11] have the most slender CB1 among ichthyosaurs examined; this makes them the least likely candidates for suction feeders among ichthyosaurs.

Lack of variation in the slenderness of the CB1 in ichthyosaurs is noteworthy. The ceratohyals of sharks show a broad range of slenderness depending on their prey capture ecology (Fig. 3B). The contrasting absence of such variation among ichthyosaurs seems to indicate that the use of the hyobranchial apparatus during feeding did not vary much among different types of ichthyosaurs, whether small or large, long- or short-snouted. We infer that the hyobranchial apparatus played a similar role during feeding across ichthyosaurian species.

The ‘ram-feeding’ hypothesis for ichthyosaurs is further strengthened by two independent pieces of morphological evidence that are rooted in the mechanics of suction feeding. First, as evident from MPCI values, syringe-like suction pressure concentration that occurs between the throat and mouth opening was not very high in ichthyosaurs, unlike in suction-feeding cetaceans. The MPCI values of ichthyosaurs are similar to those of ‘ram-feeding’ marine mammals, again suggesting that ichthyosaurs were ‘ram feeders’. One exception is *Eurhinosaurus* that is discussed later. Second, ichthyosaurian mandibles are much more elongated than those of odontocete cetaceans on average (Figs. 2 and 5B). The most elongated mandibles among suction-feeding cetaceans are found in beaked whales, which are about as acute as the least elongated mandible of ichthyosaurs. Despite the unusually elongated mandibles for a suction feeder, beaked whales are able to suction feed thanks to the presence of superficial tissues around the corners of the gape, which in effect shorten the span of gape, while allowing a more rounded opening that is suitable for concentrating pressure projection [50]. This effective decrease in gape also has an osteological basis: beaked whales have the precoronoid crest of the mandible, a unique structure that enables the mandible to overlap the upper jaw far anterior to the jaw joint. There is no evidence for such soft- or hard-tissues in ichthyosaurs.

A previous study [10] mentioned the presence of dorsally convex coronoid region in *Guanlingsaurus*. This feature, however, is not well exposed in the specimens that they described. The newer specimen reported in [16] has a complete exposure of the mandible, where no special structure analogous to the precoronoid crest of beaked whales exists. Mandibular morphology of *Guanlingsaurus* is not very different from those of some deep-jawed ichthyosaurs of the Middle and Upper Triassic except its relative shortness. Also, even if a special structure existed in the region, it probably could not shorten the gape and narrow the mouth opening effectively because of its location (see below).

A part of the reason why suction feeding did not evolve in ichthyosaurs may be the basic skull design of the Ichthyosauria, which is a clade within the Ichthyopterygia [6]. In comparison with cetaceans, the jaw joint of this clade tends to be located much more posteriorly relative to the snout, at or behind the occiput that is often inclined to contribute to the posterior displacement of the joint as in *Guanlingsaurus*. Because of this posterior location, ichthyosaurs are destined to have more elongated mandibles than cetaceans even when the snout lengths are similar. Also, this design leads to posterior location of the coronoid region in ichthyosaurs, which is usually found at the level of the occipital condyle. Thus, the region is too far away from the snout to function as the gape-shortening apparatus–note that the precoronoid crest of beaked whales is located at the posterior part of the snout. This apparent constraint from the jaw-joint location is not applicable to basal Ichthyopterygia, so it may be possible to find a suction feeder among basal members of the clade in the future.

*Eurhinosaurus* merits a separate discussion because of its uniquely low MPCI value. There is an anatomical reason why this taxon exhibits an anomalous value in this index. The genus is known for extreme shortening of the mandible, leading to an extensive overbite that is analogous to that seen in swordfish [6]. A large part of its upper dentition is anterior to the mandible, which starts to widen immediately posterior to the tip but is still not very wide at the point where the dentition ends. Apart from the overall shortening, the mandibular morphology is not unusual for an ichthyosaur. For example, the jaw symphysis is short, unlike in suction-feeding cetaceans that have low MPCI values. Given that the CB1 of *Eurhinosaurus* is slender as in other ichthyosaurs and that its hyoid corpus is unossified, it lacked the ‘equipment’ to generate suction. Therefore, it was most likely a ‘ram feeder’ as with other ichthyosaurs.

### Paleoecological Implications

Suction feeders among extant air-breathing marine vertebrates have a limited range of feeding ecology. This is partly because suction is only effective over a short distance [39], which is usually less than 6 cm in the cetaceans measured so far [35], [44]. These air-breathing suction feeders almost exclusively feed on stationary or slow-moving prey and capture them with limited pursuit. For example, suction-feeding pinnipeds and sharks feed from the sea floor [57]. With the exception of *Delphinapterus*, which use suction feeding during shallower benthic foraging [35], ‘true’ suction feeders among cetaceans feed in the mesopelagic and bathypelagic zones [58]–[60]) to catch squid and fish [61]–[63]). Most mesopelagic and bathypelagic squid and fish are considered to be slow moving [64], with the exception of some large squids [65]–[67]. Beaked whales and sperm whales tend to swim through concentrations of these prey items in the lower DSL (Deep Scattering Layer) and benthopelagic layers during feeding dives [60], [68], although sperm whales reportedly pursue large squids from time to time [68]. If some shastasaurid ichthyosaurs were suction feeders resembling beaked whales as once suggested [10], then they would have needed concentrations of slow-moving, soft-bodied prey, most likely coleoid cephalopods, as in the modern mesopelagic/bathypelagic zones. None of the benthic feeders among suction-feeding, air-breathing marine predators is edentulous except the gray whale that uses side-suction [55], so benthic feeding is probably inappropriate for these ichthyosaurs.

**Figure 6:**
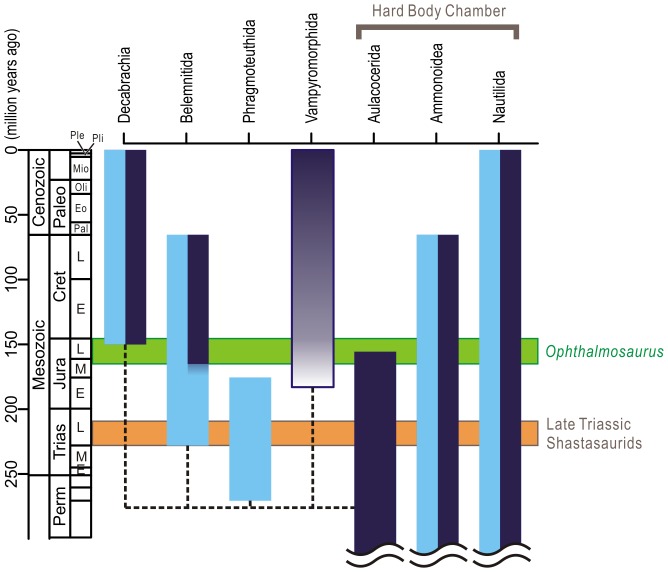
Stratigraphic ranges of major coleoid and key ichthyosaur groups being discussed. Divergence time and tree topology is based on [70]. Ranges of fossil coleoid groups are based on [79], [89]. The shastasaurid ichthyosaurs of the Late Triassic, which were previously interpreted as suction feeders resembling beaked whales [10], did not co-exist with slow-moving and soft-bodied coleoid prey suitable for such suction feeders. The deep-diving ichthyosaur *Ophthalmosaurus* was coeval with some soft-bodied coleoid vertical migrants. Dark blue indicates deep habitat (reaching the mesopelagic zone) and light blue shallow (epipelagic). The color gradation for vampyromorphs indicates uncertainty in habitat depths of early forms. The upper range of vertically migrating belemnites is extended to the level indicated by [90].

Evidence for the presence of deep-water coleoid communities in the Triassic is scant, although its possibility cannot be completely excluded. The time period predates the common ancestor of extant decabrachian coleoids in the Late Jurassic or Early Cretaceous [69], [70]–this clade has extant members that are vertical migrants [69]. The oldest belemnites are known from the Carnian of Sichuan, China [71], [72], although they did not spread worldwide until the Early Jurassic [73], [74]. This clade has evidence for vertical migration reaching at least below the mixed surface layers in the Jurassic, while most belemnites are considered epipelagic organisms [75]–[77]. The habitat depth of the only Triassic belemnites is unknown; however they appear to be shallow water forms as with most belemnites, judging from published cross-section photographs [71] (pls.III and IV) and the premises of [75]. The Triassic saw two other lineages of coleoids (Fig. 6). Aulacocerids, which spread worldwide in the Late Triassic, especially along the Tethys Sea [78], may have inhabited deepwater [75]. It has even been suggested that they were not very active [78], as expected for deepwater organisms [64], [65]. The other coleoid lineage in the Triassic, namely phragmoteuthids, had a limited geographic distribution [78] and, judging from the phragmocone angle [79] and generalization by [75], probably lived in shallower waters. Thus, aulacocerids would have been the only possible prey coleoid group for deep-diving air-breathers. At his point, it is not known if the biomass of this group was sufficiently large to support deep-diving, air-breathing predators. Moreover, aulacocerids are considered to have had an extensive shell with a ‘living chamber’ as in nautiloids but unlike modern coleoids or belemnites [80]; thus, they may not have been suitable prey of suction feeders, and certainly were not analogous to modern deepwater coleoids in terms of their characteristics as prey.

Hypothetically, it may be possible that some slow moving, soft-bodied coleoids, comparable to today's deepwater forms, inhabited shallow waters in the Triassic and could have served as potential prey for suction feeding marine reptiles. However, such a form has not been recognized to date, despite the fact that the fossil record of shallow water cephalopods is much richer than that of their deepwater counterparts. Absence of such slow moving forms in shallow water may be reasonable because the slowness of extant deepwater coleoids has been linked to the relaxed visually mediated predator/prey interactions in light-limited environments [65].

Another question is whether Triassic ichthyosaurs were capable of deep diving. Deep diving ability has been suggested for ichthyosaurs in the past but only for more derived ichthyosaurs in the Jurassic [8], [15], which had large scleral ring apertures that enabled dim-light adapted vision and a thunniform body plan that facilitated cruising ability. It is unlikely that shastasaurid ichthyosaurs were thunniform, judging from complete skeletons from China. Also, their eyes were not particularly large for ichthyosaurs, with apertures that were small for the eye size, indicating a lack of dim-light adaptation. It is thus likely that these shastasaurids were not deep divers.

Hypothetically, it may be possible that some slow-moving, soft-bodied coleoids, comparable to today's deepwater forms, inhabited shallow waters in the Triassic, providing a viable prey source for suction feeders. However, such a form has not been recognized to date, even though shallow water fossils are far more abundant than their deepwater counterparts. Absence of such shallow water forms may be reasonable because the slowness of extant deepwater coleoids has been linked to the paucity of nutrients in deep sea layers [65].

A recent study suggested that ichthyosaurian eyes evolved in response to large predators rather than deep diving [81]. However, this suggestion is not well-supported optically [82] or by fossil evidence. First, the authors mainly discussed *Temnodontosaurus* and its possible predators, whereas deep diving was suggested for *Ophthalmosaurus* and possibly other thunnosaurs that are more derived than *Temnodontosaurus* [8]. Second, *Temnodontosaurus*, when it first appeared in the Hettangian (earliest Jurassic), was by far the largest of the coeval marine reptiles despite the claim of [81]. The very large *Rhomaleosaurus* that [81] mentioned, which was comparable in body length to *Temnodontosaurus* but was much smaller in gape size than the latter, is known from the Toarcian [83], some ten million years later. Predator-driven evolution has also been proposed for deep diving in ichthyosaurs [84]; however the discussion remains qualitative, especially concerning the involvement of predators. We suggest that the availability of deepwater prey (see below) can explain the data presented in [84] as well.

The appearance of deep-diving ichthyosaurs, such as *Ophthalmosaurus*, may predate that of the common ancestors of the Decabrachia [69] (Fig. 6). However, isotopic records suggest that belemnites that were coeval with *Ophthalmosaurus* included vertical migrants [77]. Also, one belemnite that co-occurs with *Ophthalmosaurus*, namely *Cylindrotheuthis*, is thought to have been capable of withstanding shallow mesopelagic water pressure [75]. The interpretation of [75] has been challenged in the past but the basic principle of his study remains justified [85]–[88]. Additionally, coleoids belonging to the lineage of vampyromorphs are known as early as the Toarcian of the Early Jurassic [88] (Fig. 6). Although the habitat depths of these early forms are debatable, the only extant member of the lineage, vampire squid *Vampyroteuthis infernalis*, is a mesopelagic inhabitant.

## Conclusions

Triassic and Early Jurassic ichthyosaurs were most likely ‘ram-feeders’ based on functional inference from hyobranchial and mandibular morphology. Together with the inferred lack of deep-diving ability and dim-light vision in suspected suction feeders among Triassic ichthyosaurs, it is unlikely that these ichthyosaurs were meso-/bathypelagic feeders resembling beaked whales. Therefore, the evolutionary history of ichthyosaurs does not necessitate the formation of deepwater soft-bodied coleoid and fish communities in the Triassic. The coleoid fossil record also suggests the lack of deepwater coleoids with soft bodies in the Triassic. Such communities may have been available by the time *Ophthalmosaurus*, the postulated deep diving ichthyosaur, emerged. Hyobranchial morphology of marine reptiles has been largely understudied, despite its importance in inferring feeding ecology of these animals. It will be important to study it further, to understand the early evolution of modern marine ecosystems in the Mesozoic. Such a study is underway.
